# Recent Advances in NSAIDs-Induced Enteropathy Therapeutics: New Options, New Challenges

**DOI:** 10.1155/2013/761060

**Published:** 2013-09-12

**Authors:** Yun Jeong Lim, Hoon Jai Chun

**Affiliations:** ^1^Department of Internal Medicine, Dongguk University Ilsan Hospital, Dongguk University College of Medicine, 814 Siksadong, Ilsandonggu, Goyang 410-773, Republic of Korea; ^2^Department of Internal Medicine, Korea University Anam Hospital, Korea University College of Medicine, 126-1 Anam-dong 5 ga, Seongbuk-gu, Seoul 136-705, Republic of Korea

## Abstract

The injurious effects of NSAIDs on the small intestine were not fully appreciated until the widespread use of capsule endoscopy. It is estimated that over two-thirds of regular NSAID users develop injury in the small intestinal injuries and that these injuries are more common than gastroduodenal mucosal injuries. Recently, chronic low-dose aspirin consumption was found to be associated with injury to the lower gut and to be a significant contributing factor in small bowel ulceration, hemorrhage, and strictures. The ability of aspirin and NSAIDs to inhibit the activities of cyclooxygenase (COX) contributes to the cytotoxicity of these drugs in the gastrointestinal tract. However, many studies found that, in the small intestine, COX-independent mechanisms are the main contributors to NSAID cytotoxicity. Bile and Gram-negative bacteria are important factors in the pathogenesis of NSAID enteropathy. Here, we focus on a promising strategy to prevent NSAID-induced small intestine injury. Selective COX-2 inhibitors, prostaglandin derivatives, mucoprotective drugs, phosphatidylcholine-NSAIDs, and probiotics have potential protective effects on NSAID enteropathy.

## 1. Introduction

Nonsteroidal anti-inflammatory drug- (NSAID-) induced lower gastrointestinal (GI) injury is more common than NSAID-associated gastropathy [1–8]. Historically, this has been given little clinical attention since NSAID-induced enteropathy is usually asymptomatic and is not easily detected using most common diagnostic testing modalities [9, 10]. Recently, through the introduction of capsule endoscopy and device-assisted endoscopy, NSAID enteropathy has become a popular topic of study [11] particularly since NSAID enteropathy is one of the most common causes of obscure GI bleeding [11, 12]. 

Until recently, no new promising drugs have been developed for NSAID-induced enteropathy. Many efforts to determine the mechanism of NSAID-induced intestinal injury and preventive modalities have been made through experiments and clinical capsule studies (Tables 1 and 2). In this paper, we intend to review potential candidates for the prevention of NSAID-induced small intestinal injuries.

## 2. Proton Pump Inhibitors

It is not completely clear how NSAIDs damage the lower GI tract. In contrast to the stomach, there is no evidence that gastric acid plays a role in the pathogenesis of NSAID-induced lower GI injuries [13–15]. Several pharmaceutical companies are in the process of developing drugs that corelease an NSAID (naproxen, aspirin, or ibuprofen) and a proton pump inhibitor (PPI) (omeprazole, esomeprazole, or lansoprazole), and many of these compounds have already been filed with the Food and Drug Administration (FDA) or are in the late stages of development [16] (Table 3). Other companies are combining NSAIDs with high-dose H2-receptor antagonists. In both cases, these new compounds have been shown to reduce the incidence of NSAID-induced gastropathy, but not enteropathy. Moreover, there are increasing concerns about the use of PPIs in combination with aspirin or NSAIDs. Long-term use of PPIs may increase the risk of intra-abdominal infections, including spontaneous bacterial peritonitis and pseudomembranous colitis [17, 18]. Long-term use of PPIs has also been shown to cause small bowel malabsorption of certain vitamins and nutrients, resulting in osteopenia and subsequent bone fractures [19]. Many studies compared PPI use for over 5 years or longest use with last use of less than 1 year or nonuser of PPI. Long-term PPI administration worsened atrophic corpus gastritis and promoted adenocarcinoma development in Mongolian gerbils infected with *Helicobacter pylori* [20]. Additionally, PPIs may aggravate NSAID-induced intestinal injuries. Laboratory studies have shown that chronic acid suppression markedly alters the small intestinal flora, resulting in worsening of NSAID-induced enteropathy [21]. In these cases, further exploration of the potential of prebiotics and probiotics such as *Lactobacillus* to lessen these effects is warranted [21]. Unlike PPIs, the proton pump antagonist, revaprazan, did not aggravate indomethacin-induced small intestinal injuries in an animal study. However, the underlying pathophysiologic effects of this drug remain unexplained [22]. Lansoprazole was reported to have an anti-inflammatory effect through upregulation of hemeoxygenase-1, resulting in prevention of NSAID enteropathy in a rat model [23]. These results were mutually incompatible.

## 3. Cyclooxygenase-2 Inhibitor 

One of the proposed mechanisms of NSAID-induced enteropathy is impairment of mucosal defense through the inhibition of cyclooxygenase (COX), resulting in prostaglandin deficiency. Similar to the stomach, selective COX-2 inhibitors are believed to be less injurious to the small bowel than nonselective NSAIDs [24, 25]. Goldstein et al. [26] reported that a 2-week treatment course with celecoxib, a selective COX-2 inhibitor, caused fewer small intestine injuries than treatment with naproxen. Conversely, Maiden et al. [27] recently found no differences in the incidence of small intestinal injury between chronic users of nonselective NSAIDs and selective COX-2 inhibitors. While coxibs may produce less GI ulceration and bleeding than nonselective NSAIDs, they are still capable of triggering significant adverse events in the GI tract. Additionally, when given concomitantly with low-dose aspirin, the GI benefit of selective COX-2 inhibitors over nonselective NSAIDs is lost [28]. Notably, cardiovascular toxicity issues associated with highly selective COX-2 inhibitors have been a major concern, leading to the withdrawal of the highly selective COX-2 inhibitor rofecoxib (Vioxx) from the marketplace [29]. A recent large study, the Celecoxib versus Omeprazole aNd Diclofenac in Patients with Osteoarthritis and Rheumatoid Arthritis trial (CONDOR), compared the risk of injury along the entire GI tract [30]. Clinically significant decreases in hemoglobin (≥2 g/dL) and/or hematocrit (≥10% from baseline) in conjunction with a defined origin of GI bleeding were more commonly seen in patients taking diclofenac plus omeprazole rather than celecoxib (five doses or more). This clinical trial showed that celecoxib use resulted in fewer small intestinal injuries compared with a PPI plus nonselective NSAID although it is unclear whether selective COX-2 inhibitors truly prevent NSAID-associated enteropathy. Further large-scale studies are needed to determine whether the use of selective COX-2 inhibitors can abolish toxicity along the entire GI tract.

## 4. Prostaglandins

It is important to note that even when intestinal prostaglandin synthesis is markedly suppressed, ulceration and bleeding do not necessarily occur. However, exogenous prostaglandins have been shown to attenuate NSAID-induced intestinal damage. Bjarnason et al. [38] demonstrated a significant reduction in NSAID-induced intestinal permeability with use of misoprostol, but whether or not a reduction in permeability translates into a reduction in clinically significant injury is unclear. Fujimori et al. [31] demonstrated the benefits of misoprostol treatment in a pilot study in which small intestinal damage was assessed by capsule endoscopy. In that study, misoprostol cotherapy reduced the incidence of lesions in the small intestine induced by a 2-week course of diclofenac sodium in healthy subjects. Watanabe et al. [32] studied the therapeutic effects of misoprostol on aspirin-induced intestinal injury. Their subjects included patients with gastric ulcers who were taking low-dose enteric-coated aspirin by mouth. These patients were treated with a PPI for 8 weeks, at the end of which all patients exhibited redness and erosions in the small intestine by capsule endoscopy. Misoprostol was subsequently administered instead of a PPI for an additional 8 weeks, after which the small intestinal lesions had improved on follow-up capsule endoscopy. Thus, misoprostol showed the ability to induce healing of aspirin-induced small bowel injury. Use of misoprostol has unavoidable limitations, as it causes well-known GI side effects such as diarrhea, abdominal pain, dyspepsia, and nausea. 

## 5. Cytoprotective Drugs

Rebamipide is a cytoprotective drug that induces the production of intracellular prostaglandins, improves blood flow, blocks increases in permeability, scavenges free radicals, and ultimately exhibits anti-inflammatory properties [39]. Two prospective, double-blind studies with capsule endoscopy using rebamipide in healthy subjects had been taken [33, 34]. When the subjects received a placebo, there were significantly more NSAID-induced mucosal injuries in the small intestine compared with when they received rebamipide. A similar randomized, placebo-controlled, double-blind, crossover study focused on the effects of 4 weeks of low-dose aspirin ingestion on small bowel damage using capsule endoscopy in healthy subjects [35]. In this study, long-term, low-dose aspirin use induced small bowel damage, while rebamipide prevented this damage. Thus, rebamipide may be a candidate drug for preventing aspirin-induced small bowel injury. However, these studies were limited by their small sample sizes. Another cytoprotective drug, geranylgeranylacetone (also known as teprenone), was reported to protect against diclofenac-induced injuries in the small intestine in a small-scale clinical trial using capsule endoscopy [36].

## 6. Antimicrobials

Gram-negative bacteria are important in the pathogenesis of NSAID enteropathy [40, 41]. Administration of NSAIDs to rodents has been shown to cause profound changes in the composition of enteric bacteria, resulting in the development of ulcers in the small intestine [40–42]. It has also been reported that treatment with broad-spectrum antibiotics can reduce the severity of NSAID enteropathy; one study showed that germ-free rats and mice do not develop NSAID enteropathy [43]. In that study, germ-free mice were susceptible to NSAID enteropathy when colonized with *Escherichia coli* or *Eubacterium limosum*, but not when colonized with probiotics such as *Bifidobacterium adolescentis* or *Lactobacillus acidophilus*. NSAIDs invade the intestinal mucosa and activate Toll-like receptor, which is also activated by the lipopolysaccharides found on Gram-negative bacteria [44]. Toll-like receptor has been known to play a key role in a variety of intestinal injuries via stimulation of inflammatory responses. 

It is suggested that metronidazole is potentially beneficial in preventing NSAID-induced intestinal injuries by reducing intestinal permeability and inflammation [45]. 

## 7. New Compounds (Table 3)

Bile is an important factor in the pathogenesis of NSAID enteropathy [40]. Ligation of the bile duct to prevent enterohepatic recirculation of NSAIDs prevents intestinal damage [46]. While NSAIDs themselves can cause damage to intestinal epithelial cells, disruption of the lipid bilayer of epithelial cells and other damaging effects are enhanced when NSAIDs are combined with bile [47]. This cytotoxic action of NSAIDs in combination with natural bile and/or synthetic bile acids can be prevented with the addition of phosphatidylcholine (PC) [48]. PC-NSAIDs have been developed by PLxPharma (Houston, TX, USA) [48, 49], and one animal study showed that PC indomethacin does not induce small intestinal injuries [22]. Mucosal surfactant phospholipids form a nonwettable, hydrophobic lining that limits the entrance of acid, bile, and other irritants [49]. Preassociating NSAIDs with exogenous PC prevented an increase in surface wettability and protected GI mucosa against the injurious side effects of NSAIDs [50, 51]. Extensive animal studies have demonstrated that PC-NSAIDs offer a lower risk of GI erosion and ulceration while maintaining the pharmacological activity and bioavailability of parent NSAIDs. In one clinical trial, ibuprofen PC was shown to be an effective osteoarthritic agent with an improved GI safety profile compared with ibuprofen alone in older (>55 years) patients, who are more susceptible to NSAID-induced gastroduodenal injury [52].

Other attempts have been made to develop new compounds such as nitric oxide (NO) NSAIDs and hydrogen sulfide (H_2_S) NSAIDs with the intention of improving GI tolerability [53]. NO may exert its protective effects on the GI mucosa by maintaining the defense mechanisms that are disrupted by COX inhibitors such as mucosal blood flow and mucus and bicarbonate secretion [53, 54]. In addition, NO decreases neutrophil-endothelial adherence, which plays an important role in NSAID-induced GI mucosal injury. Awareness of the protective effects of NO has led to the development of a novel class of drugs called cyclooxygenase-inhibiting NO donors (CINODs). In animal studies, CINODs produced an increase in plasma nitrite/nitrate levels and marked reductions in gastroduodenal and small bowel injuries [54]. While NO can exert both proinflammatory and anti-inflammatory effects, CINODs are generally known to exhibit enhanced anti-inflammatory activity. However, it is still unclear whether CINODs can improve total GI tolerability. 

H_2_S is an endogenous gaseous mediator that suppresses leukocyte adherence to the vascular endothelium and inhibits proinflammatory cytokine synthesis [55, 56]. It has been reported that H_2_S donors can increase the resistance of the gastric mucosa to injury and accelerate the healing of preexisting ulcers [55]. These observations suggest that NSAIDs that have been modified to release H_2_S will exhibit reduced toxicity [56]. H_2_S-releasing NSAIDs, including derivatives of naproxen, diclofenac, aspirin, and indomethacin, have been synthesized and shown to have markedly improved efficacy and reduced toxicity compared with the corresponding parent anti-inflammatory drugs [57]. It is not clear whether H_2_S-releasing NSAIDs can also improve lower GI tolerability.

## 8. Probiotics

Several researchers have evaluated the role of probiotics against indomethacin or aspirin enteropathy *in vitro* and in animal models [37, 58–61]. Their results were mutually incompatible. Double-blind, crossover, placebo-controlled studies have been done to evaluate the protective effects of probiotics [37, 60, 61]. In one study, *Lactobacillus casei* treatment was shown to reduce small bowel injury based on capsule endoscopic findings in chronic low-dose aspirin users [37]. However, evidence regarding the protective roles of probiotics is still weak. Larger, well-designed studies using different probiotic strains, optimal doses, and durations are necessary to clarify their roles. 

## 9. Conclusions 

NSAID-induced enteropathy is common and reported the incidence of intestinal damage up to two-thirds [1–6]. However, NSAID-induced small intestinal lesions did not cause the clinical outcomes including perforation, obstruction, and bleeding on every hedge. It is not clearly beneficial to prevent NSAID-induced small intestinal lesions, for example, erosions, red spots, or denuded area. However, NSAID-induced lower GI complications (perforation, bleeding, or obstruction) are increasing while upper GI complications are decreasing [9, 62]. Lower GI events accounted for 40% of all serious GI events in patients on NSAIDs [25]. Although it is generally not recommended in naïve NSAID users, we should prevent NSAID-induced lower GI injuries in persons with a prior history of NSAID-induced clinically significant lower GI events. Selective COX–2 inhibitors, prostaglandin derivatives, cytoprotective drugs, PC-NSAIDs, and probiotics have all been shown to have potential protective effects on NSAID-induced small intestinal injuries. Future directions include the development of an NSAID compound with total (upper and lower) GI tract tolerability and inappreciable cardiovascular toxicity.

## Figures and Tables

**Table 1:** Proposed pathophysiologic mechanism and protection of NSAID-induced small intestinal injuries.

Cause	Protection	Advantages	Weak points
Cyclooxygenase inhibition	COX-II inhibitor	Protection of upper and lower GI complications contrary to PPI + nonselective NSAID	No beneficial effect on user of selective COX-2 inhibitors for over 3 months
Decreased prostaglandin synthesis	Prostaglandins	Preventive effect on clinical studies using capsule endoscopies	Lower compliance due to side effects (diarrhea, abdominal pain, dyspepsia, etc.)
Gram-negative bacteria	Antimicrobials	Strong experimental studies high light that Gram-negative bacteria play an important role in NSAID enteropathy	No well-designed clinical studies
Bile	Phosphatidylcholine-NSAID	Cytotoxic action of NSAID in combination with bile acids in preclinical studies	No well-designed clinical studies
Dysbiosis	Probiotics	Positive results on preclinical and clinical studies	Optimal dose of each strains is not determined

COX: cyclooxygenase.

**Table 2:** Clinical trials using capsule endoscopy about protective effect on NSAID-induced small intestinal injuries.

Author (year)	Protective drug	NSAID	Period
Goldstein et al. [26] (2005)	Celecoxib	Naproxen	2 weeks
Fujimori et al. [31] (2009)	Misoprostol	Diclofenac sodium	2 weeks
Watanabe et al. [32] (2008)	Misoprostol	Aspirin (low dose)	8 weeks
Niwa et al. [33] (2008)	Rebamipide	Diclofenac	1 week
Fujimori et al. [34] (2011)	Rebamipide	Diclofenac	2 weeks
Mizukami et al. [35] (2011)	Rebamipide	Aspirin (low dose)	4 weeks
Niwa et al. [36] (2009)	Geranylgeranylacetone	Diclofenac sodium	1 week
Endo et al. [37] (2011)	*Lactobacillus casei *	Aspirin + omeprazole	3 months

**Table 3:** New hybrid compounds.

	Representative composition	Advantages	Stage
PPI	Esomeprazole + naproxen Esomeprazole + aspirin Lansoprazole + naproxen	Protection of NSAID gastropathy	FDA approval
H_2_ blocker	Famotidine + ibuprofen	Protection of NSAID gastropathy	FDA approval
NO	NO releasing naproxen	Improved cardiovascular toxicity	Preclinical trial
H_2_S	H_2_S releasing naproxen	Improved cardiovascular toxicity	Preclinical trial
Phosphatidylcholine	Phosphatidylcholine-ibuprofen Phosphatidylcholine-aspirin	Protective effect on NSAID-induced small intestinal injuries in animal study	Clinical trial
Dimethyl sulfoxide	Diclofenac in dimethyl sulfoxide	Dermal administration	FDA approval

PPI: proton pump inhibitor; NSAID: nonsteroidal anti-inflammatory drug; FDA: Food and Drug Administration.
